# Serum protein mediators of dementia and aging proper

**DOI:** 10.18632/aging.101091

**Published:** 2016-12-03

**Authors:** Donald R. Royall, Safa Al-Rubaye, Ram Bishnoi, Raymond F. Palmer

**Affiliations:** ^1^ Department of Psychiatry, University of Texas Health Science Center at San Antonio, San Antonio, TX 78229, USA; ^2^ Department of Medicine, University of Texas Health Science Center at San Antonio, San Antonio, TX 78229, USA; ^3^ Department of Family and Community Medicine, University of Texas Health Science Center at San Antonio, San Antonio, TX 78229, USA; ^4^ South Texas Veterans’ Health System Audie L. Murphy Division GRECC, San Antonio, TX 78229, USA; ^5^ Department of Psychiatry, The Medical College of Georgia, Augusta, GA 30912, USA

**Keywords:** aging, cognition, dementia, functional status, *g*, intelligence

## Abstract

The latent variable “δ” (for “dementia”) appears to be uniquely responsible for the dementing aspects of cognitive impairment. Age, depressive symptoms, gender and the apolipoprotein E (APOE) ε4 allele are independently associated with δ. In this analysis, we explore serum proteins as potential mediators of age's specific association with δ in a large, ethnically diverse longitudinal cohort, the Texas Alzheimer's Research and Care Consortium (TARCC). 22 serum proteins were recognized as partial mediators of age's association with δ. These include Insulin-like Growth Factor-Binding Protein 2 (IGF-BP2), which we had previously associated with age-specific cognitive change, and both Pancreatic Polypeptide (PP) and von Willebrand Factor (vWF), previously associated with δ. Nine other δ-related proteins were not confirmed by this ethnicity adjusted analysis. Our findings suggest that age's association with the disabling fraction of cognitive performance is partially mediated by serum proteins, somatomedins and hormones. Those proteins may offer targets for the specific treatment of age-related effects on dementia severity and conversion risk.

## INTRODUCTION

Age, depression, and the apolipoprotein E (APOE) ε4 allele are independently associated with the latent dementia phenotype “δ” (for “dementia”) [1]. Their associations with dementia do not necessarily involve neurodegeneration. Depression's association with cognitive decline in older persons is not mediated by neurodegenerative changes [2], while age's association with δ has been shown to be fully mediated by *a paucity* of neurodegenerative changes in pathologically confirmed Alzheimer's Disease (AD) cases [3]. Brain aging is therefore not AD [4].

On the other hand, clinical “AD” may very well have an aging component. Since δ is essentially the sole cognitive determinant of dementia severity, clinical dementia must arise from the sum of all independent δ-related processes. Age's small independent effect appears to be linear over the lifespan, and cumulative [5]. Over a 50 year age range, aging might account for up to a standard deviation change in composite “d-scores”. That is not trivial. δ's intercept and slope are uniquely strong determinants of future dementia status [6-7]. Each quintile in the d-score distribution of non-demented persons increases conversion to clinical “AD” by 50% [three-fold among “Mild Cognitive Impairment (MCI)” cases] [8].

In the “oldest old”, aging alone might sum with comorbid neurodegenerative processes to push d-scores into their demented range. This should effectively reduce the amount of neuropathology required to make the diagnosis of dementia in centenarians, and modulate the apparent associations between various neuro-pathologies and clinical dementia. In fact, dementia at advanced age is associated with lower levels of AD-specific neuropathology [9], and less widely spread pathology [10].

Even in their aggregate, demographic-specific dementia risks explain a minority of δ's variance [1]. Thus, regardless of whether age's effect is mediated by neurodegeneration, observed dementia status must be largely determined by age-independent factors. In Age, depression and APOE adjusted models, we have found the majority of δ's remaining variance to be associated with a large number of pro- and anti-inflammatory serum protein biomarkers [1, 11-13].

On the other hand, we have reported serum Insulin-like Binding Protein 2 (IGF-BP2) to be a strong correlate of age's specific cognitive effects [14]. However, age has both direct (δ-independent) and indirect (δ-related) effects on cognition. It has yet to be determined whether IGF-BP2 mediates age's association with δ, or its δ-independent direct effects instead.

In this analysis, we combine structural equation models (SEM) with longitudinal data from the Texas Alzheimer's Research and Care Consortium (TARCC) to explore more than 100 serum proteins as potential mediators of age's specific association with δ. Our models are constructed such that the significant mediators of age's effect on prospective δ scores can be interpreted causally. The mediators should offer both insights into the pathophysiology of Aging Proper, and potential targets for the remediation of age-specific cognitive impairments.

## RESULTS

The demographic characteristics of our sample are presented in Table 1. The ethnicity equivalent unadjusted Visit 2 δ homolog composite score (i.e., “dEQ”) achieved a high Area Under the Receiver Operating Characteristic curve (AUC/ROC) for the discrimination between AD cases and normal controls (NC) (AUC = 0.953; CI: 0.946-0.960). “g’”'s (i.e., δ's residual in Spearman's general intelligence factor “*g*”) AUC for the same discrimination was at a near chance level [AUC = 0.536 (CI: 0.514-0.558)]. This is consistent with past findings, across batteries, in this and other cohorts.

**Table 1:** Descriptive Statistics

Variable	N	Mean (SD)
**Age** (observed)	3381	70.88 (9.48)
**APOE e4 alleles** (1 = e4+, n = 1223)	3154	0.39 (0.49)
**CDR (Sum of Boxes)**	3306	2.42 (3.35)
**COWA**	3381	8.41 (3.49)
**DIS**	3381	8.89 (3.01)
**EDUC** (observed)	3381	13.24 (4.25)
**Ethnicity** (1 = MA, n = 1189)	3381	0.36 (0.47)
**GDS_30_**(observed)	3005	5.60 (5.25)
**Gender** (♂ = 1, n = 1281)	3312	0.39 (0.49)
**IADL (Summed)**	3381	10.48 (4.52)
**MMSE**	3311	25.52 (4.76)
**WMS LM II**	3381	8.05 (4.30)
**WMS VR I**	3381	7.88 (3.68)
**Complete Cases**	2861	

CDR = Clinical Dementia Rating scale; COWA = Controlled Oral Word Association Test; DIS = Digit Span Test; GDS = Geriatric Depression Scale [66]; IADL = Instrumental Activities of Daily Living; MMSE = Mini-Mental State Exam [67]; SD = standard deviation; WMS LM II = Weschler Memory Scale: Delayed Logical Memory; WMS VR I = Weschler Memory Scale: Immediate Visual Reproduction.

The Base Model had excellent fit [χ^2^ = 5.59 (13), p = 0.960; CFI = 1.00; RMSEA = 0.00]. Independently of the covariates [i.e., APOE ε4 allelic burden, depressive symptoms, education, ethnicity, gender, homocysteine (HCY), and hemoglobin A1c (HgbA1c)], baseline age was significantly directly associated with Visit 2 dEQ (r = −0.25, p<0.001), and weakly with the Visit 2 g’ composite (r = −0.11, p ≤ 0.001). Age's significant association with Visit 2 dEQ scores was in a negative direction suggesting an adverse effect on observed cognitive performance.

**Figure 1.**
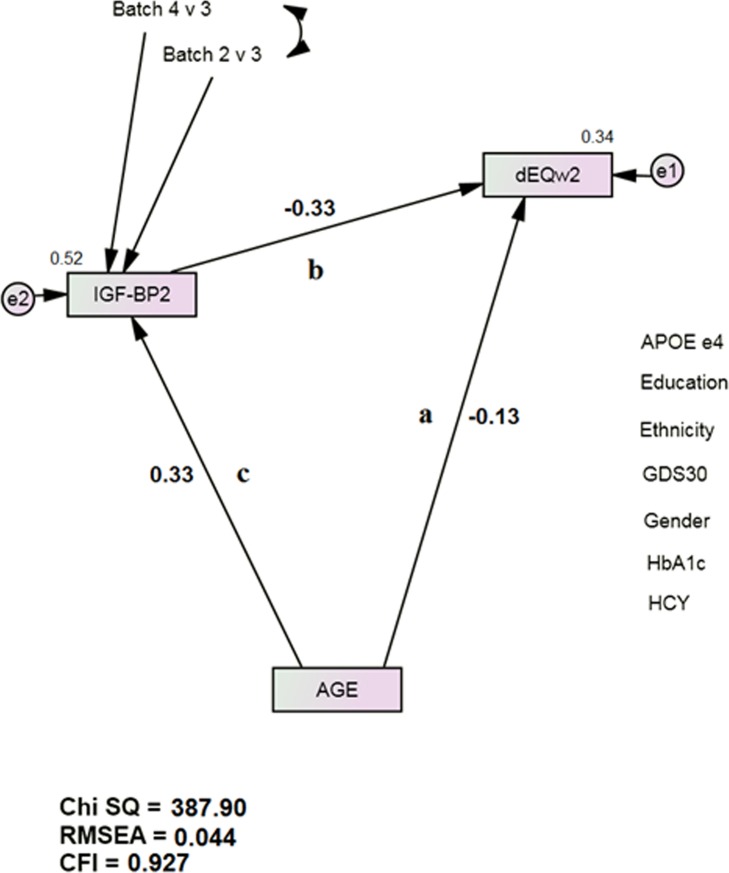
IGF-BP2 Mediates Age's Direct Association with Future Dementia Severity, as measured by dEQ APOE = apolipoprotein e4 status; CFI = Comparative Fit Index; GDS = Geriatric Depression Scale; HCY = serum homocysteine; HgbA1c = serum hemoglobin A1c; IGF-BP2 = Insulin-like Growth Factor Binding Protein 2; RMSEA = Root Mean Square Error of Association. *All observed variables except AGE are adjusted for APOE, education, ethnicity, gender, GDS, HCY, and HgbA1c (paths not shown for clarity). The covariates are densely intercorrelated.

The mediation models all had acceptable fit [e.g., IGF-BP2: χ^2^ = 387.90 (17), p < 0.001; CFI = 0.927; RMSEA = 0.044 (Figure 1)]. 22 proteins achieved statistically significant mediation effects after Bonferroni correction for multiple comparisons (Table 2). IGF-BP2 had previously been recognized as an age-specific serum protein biomarker [14]. Pancreatic Polypeptide (PP) and von Willebrand Factor (vWF) had previously been recognized as δ-related serum protein biomarkers [13]. Table 3 presents the mediation effects. All the identified proteins were partial mediators, but several had relatively large effects (range 9.9 – 45.2%). We did not test multivariate mediations or interactions.

**Table 2:** Potential Mediators of Age's-Specific Dementing Effect

Adiponectin
(APN)Angiopoetin-2N (ANG-2)
Compliment 3 (C3)
Creatinine Kinase-MB (CK-MB)
Epidermal Growth Factor Receptor 1 (EGFR)
FAS
Follicle stimulating hormone (FSH)
Glutathione S-Transferase (GST)
granulocyte colony stimulating factor (G-CSF)
Insulin-like Growth Factor-1 (IGF-I)
Insulin-like Growth Factor-Binding Protein 2 (IGF-BP2)*
Interleukin 5 (IL-5)
Myoglobin (MyG)
Pancreatic Polypeptide (PP) ^†^
Plasminogen Activator Inhibitor type 1(PAI-1)
Platelet-Derived Growth Factor (PDGF)
Progesterone
Resistin
S100b
Serum Amyloid P (SAP)
Thyroxine Binding Globulin (TBG)
von Willebrand Factor (vWF)^†^

* Previously recognized aging biomarker [14].

† Previously recognized biomarker of δ [13].

**Table 3:** Mediation Effects (Class 1)

Mediating Biomarkers	Adjusted Path a (Figure 1)	Z (p)	Effect (%)
Adiponectin (APN)	−0.22, p < 0.001	−4.26 (<0.001)	11.7
Angiopoetin-2 (ANG-2)	−0.20, p < 0.001	−4.72 (<0.001)	13.8
Compliment 3 (C3)	−0.27, p < 0.001	4.46 (<0.001)	11.9
Creatinine Kinase-MB (CK-MB)	−0.21, p < 0.001	−5.50 (<0.001)	13.1
Epidermal Growth Factor Receptor 1 (EGFR)	−0.19, p < 0.001	−6.15 (<0.001)	22.5
Fatty Acid Synthase (FAS)	−0.22, p < 0.001	−2.65 (0.004)	14.1
Follicle stimulating hormone (FSH)	−0.20, p < 0.001	−5.27 (<0.001)	14.0
Glutathione S-Transferase (GST)	−0.28, p < 0.001	3.80 (<0.001)	17.2
granulocyte colony stimulating factor (G-CSF)	−0.21, p < 0.001	−5.22 (<0.001)	12.6
Insulin-like Growth Factor-1 (IGF-I)	−0.25, p < 0.001	1.83 (0.03)	9.9
Insulin-like Growth Factor-Binding Protein 2 (IGF-BP2)	−0.13, p < 0.001	−8.85 (<0.001)	45.2
Interleukin 5 (IL-5)	−0.27, p < 0.001	2.57 (0.005)	17.6
Myoglobin (MyG)	−0.30, p < 0.001	4.87 (<0.001)	21.6
Pancreatic Polypeptide (PP)	−0.21, p < 0.001	−4.71 (<0.001)	14.1
Plasminogen Activator Inhibitor type 1(PAI-1)	−0.21, p < 0.001	−5.50 (<0.001)	15.0
Platelet-Derived Growth Factor (PDGF)	−0.29, p < 0.001	4.22 (<0.001)	17.8
Progesterone	−0.27, p < 0.001	2.56 (0.005)	12.2
Resistin	−0.20, p < 0.001	−4.39 (<0.001)	13.8
S100b	−0.28, p < 0.001	4.97 (<0.001)	18.1
Serum Amyloid P (SAP)	−0.21, p < 0.001	−5.66 (<0.001)	13.5
Thyroxine Binding Globulin (TBG)	−0.22, p < 0.001	−4.60 (<0.001)	9.9
von Willebrand Factor (vWF)	−0.22, p < 0.001	−3.92 (<0.001)	11.0

Table 4 presents other age-related proteins, unrelated to δ by path b (Figure 1). Alpha2-macroglobulin (α2M), Interferon-gamma (IFN-γ), Interleukin 10 (IL-10), Interleukin 12-p40 (IL-12p40), Interleukin 15 (IL-15), Prolactin (PRL), Stem Cell Factor (SCF), Thrombopoietin (THPO), and Tumor Necrosis Factor alpha (TNF-α) had previously been associated with δ in non-Hispanic White (NHW) TARCC participants [1, 13]. None were associated with δ in these ethnicity adjusted models.

Table 5 presents δ-related proteins unrelated to age by path c (Figure 1). Beta2-Microglobulin (β2M) was the only previously recognized δ biomarker [13]. The remainders are newly recognized as such. Table 6 lists biomarkers that were related neither to age nor to δ.

**Table 4:** Other Age-Related Biomarkers (unrelated to the dEQ by Path b)

Adrenocorticotropic Hormone (ACTH)
alpha1-antitrypsin (A1AT)
alpha2-macroglobulin (α2M)*
alpha-Fetoprotein (α-FP)
Amphiregulin (AREG)
Angiotensinogen
AXL
Betacellulin
Bone Morphogenic Protein 6 (BMP6)
Cortisol
Eotaxin-3
Epiregulin (EREG)
FAS-Ligand (FAS-L)
Heparin-binding EGF-like growth factor (HB-EGF
Hepatocyte Growth Factor (HGF)
Interferon-gamma (IFN-γ)*
Interleukin 1 receptor (IL-1r)
Interleukin 3 (IL-3)
Interleukin 7 (IL-7)
Interleukin 10 (IL-10)*
Interleukin 12-p40 (IL-12p40)*
Interleukin 13 (IL-13)
Interleukin 15 (IL-15)^††^
Interleukin 16 (IL-16)
Lipoprotein a Luteinizing Hormone (LH)
Matrix Metalloproteinase type 3 (MMP-3)
Prolactin (PRL)*
Prostatic Acid Phosphatase (PAP)
Pulmonary and Activation-Regulated Chemokine (PARC)
Serum Glutamic Oxaloacetic Transaminase (SGOT)
Stem Cell Factor (SCF) *
Thrombopoietin (THPO)*^†^
Thrombospondin-1 (THBS1)Thymus-Expressed Chemokine (TECK)
Tissue Factor (TF)
Tissue Growth Factor alpha (TGF-α)
Tissue Inhibitor of Metalloproteinase type 1 (TIMP-1)
Tumor Necrosis Factor alpha (TNF-α)*
Tumor Necrosis Factor-Related Apoptosis-Inducing Ligand Receptor 3 (TRAIL-R3)
Vascular Endothelial Growth Factor (VEGF)‡

* Previously recognized δ biomarkers in non-Hispanic Whites (NHW) only [13],

† [1] (i.e., unconfirmed as a biomarker of dEQ in this ethnicity adjusted analysis).

†† Previously recognized ethnicity adjusted δ biomarker [11].

‡ δ-related trend, p = 0.002.

**Table 5:** Age-independent dEQ Biomarkers (unrelated to Age by Path c)

beta2-Microglobulin (β2M)*
Brain-Derived Neurotrophic Factor (BDNF)
Carcinoembryonic antigen (CEA)
CD40
Chromogranin A
Fatty Acid Binding Protein (FABP)
Growth Hormone
Immunoglobulin M (IgM)
Insulin
Interleukin 8 (IL-8)
Interleukin 18 (IL-18)
Macrophage Inflammatory Protein type 1 alpha (MIP-1α)
RANTES
Sex Hormone Binding Globulin (SHBG)
Tenascin C
Testosterone
Tumor Necrosis Factor receptor type II (TNF-RII)
Vascular Cell Adhesion Molecule type 1 (VCAM-1)

* Previously recognized δ biomarker [13].

**Table 6:** Unrelated Biomarkers

Angiotensin Converting Enzyme (ACE)
Agouti-Related Protein (AgRP)
Apolipoprotein A1 (APOA1)
Apolipoprotein CIII (APOCIII)
Apolipoprotein H (apoH)
B Lymphocyte Chemoattractant (BLC)
Cancer Antigen 125 (CA 125)
Cancer Antigen 19-9 (CA 19-9)
CD40 Ligand
Connective Tissue Growth Factor (CTGF)
C Reactive Protein (CRP)
ENA-78 (ENA-78)
EN-RAGE (EN-RAGE)
Epidermal Growth Factor (EGF)
Eotaxin
Factor VII
Ferritin
Fibrinogen
GRO alpha (GROa)
Haptoglobin
Human CC Cytokine (HCC-4)
I-309
Immunoglobulin A (IgA)
Immunoglobulin E (IgE)
Intercellular Adhesion Molecule, type 1 (ICAM-1)
Interleukin 1 receptor antagonist (IL-1ra)
Leptin
Macrophage Inflammatory Protein type 1 beta (MIP-1b)
Macrophage Derived Chemokine (MDC)
Macrophage Migration Inhibitory Factor (MMIF)
Monocyte Chemotactic Protein type 1 (MCP-1)
Prostate Specific Antigen (PSA)
Serum Amyloid P (SAP)
Soluble Advanced Glycosylation End Product-Specific Receptor) (sRAGE)
Sortilin
Thyroid Stimulating Hormone (TSH)
Tumor Necrosis Factor beta (TNFb)
Vitamin D Binding Protein (VDBP)†

† Previously recognized δ biomarker (Bishnoi, Palmer & Royall, 2015).

## DISCUSSION

We have surveyed more than 100 potential mediators of age's specific and significant association with the latent dementia phenotype, δ. Our sample size was large, and we were powered to detect even statistically weak effects. All our findings have been replicated in random subsets of TARCC's data. We have replicated our previously reported association between age and IGF-BP2, and three of our previously observed age-independent associations with δ, even though 1) TARCC's sample size has increased over time, 2) we are using a new δ homolog, 3) the biomarkers are being used here to predict future cognitive performance, and 4) the prior associations were obtained using raw biomarker data, while these employ normalized variables.

We have identified four classes of proteins: 1) potential mediators of age's significant direct effect on δ, 2) δ-independent age-related proteins, 3) age-independent predictors of δ, and 4) proteins related neither to age nor to δ.

While many proteins were related to age, only a subset was also associated with δ (Class 1, Table 2). δ in turn has been associated with atrophy in the Default Mode Network (DMN) [15]. This suggests that the mediators in Table 2 may effect aging-specific changes to the structure or function of the DMN.

The DMN is a network of interconnected brain regions that are particularly active in the resting state [16]. Functional connectivity studies in older subjects have shown decreased DMN connectivity [17-20] and less deactivation during task performance [21-22]. The impact of aging-related serum biomarkers on the integrity and functioning of the DMN is not well-studied. However, Thompson et al. [23] found that elevated Serum protein S100B levels significantly correlated with DMN activity. S100B has been confirmed by this analysis to mediate age's specific-effect on a DMN-related cognitive construct (i.e., δ).

Our observations may help further clarify age's specific effects on cognitive function. First, although age has both direct and indirect effects on observed cognitive performance [5], only its indirect effects, mediated by δ, are functionally salient, and thus “dementing.” This constrains “senility” and its biology to an effect on intelligence.

Second, δ has been shown to be “agnostic” to dementia's etiology [6]. Age's association with δ suggests that it too may have a role in determining *all cause* dementia risk, not just AD risk. This risk may not be conveyed through neurodegeneration. Age's specific association with δ is characterized by lesser levels of AD-specific lesions [3].

Age accounts for only 5% of δ's variance in this sample (per the base model). Regardless, correcting any δ-related pathology might improve dementia status, including age's small effect. The mediators identified in Table 2 then, may offer targets for the remediation of age's specific contribution.

Each Class 1 protein is a partial mediator of age's contribution, ranging from Thyroxine Binding Globulin (TBG) (9.9%) to IGF-BP2 (45.2%)(Table 3). In their aggregate, they may have interacting effects. For example, S100b is elevated after cardiac surgery and correlated with post-operative cognitive impairments [24]. It binds to the receptor for advanced glycation end products (RAGE), which induces nuclear factor kappa-B (NF-kappaB)-regulated cytokines, including Compliment 3 (C3) [25]. However, we did not test multivariate interactions.

We note that not all of the mediators attenuate age's direct effect. C3, Glutathione S-Transferase (GST), Interleukin 5 (IL-5), Myoglobin (MyG), Platelet-Derived Growth Factor (PDGF), Progesterone, and S100b accentuated age's adverse effect on δ.

We had previously identified IGF-BP2 as a strong predictor of a δ ortholog targeting age itself instead of IADL [14]. That ortholog was significantly associated with δ, suggesting overlap between δ and Aging Proper. It has since been shown that δ mediates the majority of age's effect on cognition, but not all [5]. IGF-BP2's appearance in Class 1 confirms its contribution to age's dementing aspect (i.e., “Senility”).

Class 1 also contains Insulin-like Growth Factor-1 (IGF-1). The appearance of both IGF-1 and IGF-BP2 among the Class 1 mediators strongly implicates the insulin-like growth factor (IGF) system in Aging Proper. The IGF system is comprised of two growth factors (IGF-I and 2), six high affinity binding proteins (IGF-BP 1 to 6) and four receptors [26-27]. Most of these are not available in TARCC.

Serum levels of IGFs I and II appear to mediate growth hormone (GH)-related somatotrophic changes in humans. These “somatomedins” circulate in non-covalent associations with IGF-BP2. It has been suggested that decreased function of the GH-somatomedin axis is responsible for age-specific anabolic changes (e.g., the “somatopause”)[28]. Interestingly, GH itself appears to be an age-independent δ-related protein (Class 3) (Table 5).

Serum IGF-BP2 increases with age, and high serum levels have been associated with greater disability, poorer physical performance, reduced muscle strength and lower mineral bone density [29]. Serum IGF-I declines with age [30-31]. Consistent with those findings, IGF-BP2's association with age was positive (Figure 1) while IGF-I's association with age was inverse. Insulin itself is related to δ, but not to age (in this HgbA1c adjusted analysis) (Table 5).

MyG and Creatinine Kinase-MB (CK-MB)'s appearance among the Class 1 mediators, recent associations between simple motor tasks and dementia risk [32], and the age-related somatomedins in Classes 1 and 2 lend credence to the hypothesis that there is a cognitive ortholog of somatic “frailty” [33].

IGF-I had an inverse adverse effect on dEQ. Other adverse mediators were Adiponectin (APN), Angiopoetin-2N (ANG-2), C3, CK-MB, Fatty Acid Synthase (FAS), Follicle stimulating hormone (FSH), Glutathione S-Transferase (GST), IGF-BP2, PP, PDGF, Progesterone, Resistin, and vWF. Like IGF-1, C3, GST, PDGF, and Progesterone declined with age [while also accentuating age's effect on dEQ (see above)]. The others increased significantly with age. Thus all might contribute to age's adverse effect on δ.

Epidermal Growth Factor Receptor 1 (EGFR), granulocyte colony stimulating factor (G-CSF), IL-5, MyG, Plasminogen Activator Inhibitor type 1(PAI-1), S100b, Serum Amyloid P (SAP) and Thyroxine Binding Globulin (TBG) had positive associations with δ and might offer some protection from age's otherwise adverse effects.

The mechanism(s) by which the other Class 1 proteins affect δ remain to be elucidated. However, aging's pathophysiology will be necessarily constrained, by δ's mediation of its dementing effects, to the physiological processes that mediate intelligence. Two candidate processes might be synaptogenesis and network connectivity. C3, IGF-I, Progresterone, PAI-1, and S100b, all Class 1 mediators, are modulators of synaptic structure and function [34-38].

Class 2 (Table 4) comprises proteins that although age-related, never the less fail to contribute to dementia via δ scores. They may mediate non-dementing age-related cognitive changes via *g*'s “domain-specific” residuals (e.g., memory, etc.). Alternatively, they may contribute to Aging Proper's manifestation in other tissues or organs.

Notable among these are multiple EGFR agonist ligands, including Amphiregulin (AREG), Betacellulin, Epiregulin (EREG), Heparin-binding EGF-like growth factor (HB-EGF), and Tissue Growth Factor alpha (TGF-α) [39]. Epidermal Growth Factor (EGF), another EGFR agonist ligand, showed statistically insignificant trends as a potential mediator. The EGFR itself is a Class 1 Mediator (Table 2). These findings suggest the EGFR family of agonist ligands may have potential roles as therapeutic agents for age-specific cognitive and /or somatic decline.

On the other hand, several EGFR antagonists are approved by the Federal Drug Administration (FDA) for the treatment of certain cancers. These might be expected to have adverse effects, according to our findings. Chemotherapy has been noted to adversely impact connectivity in the DMN [40]. Such effects might explain reports of disability due to “chemobrain” in the literature [41]. They also illustrate the potential for reciprocal relationships between cognitive performance and cancer risks. It has been suggested both that chemotherapy is a risk factor for cognitive decline in late life [42], and that AD cases are relatively protected from cancer [43].

Class 2 also includes almost all of TARCC's interleukin panel. The interleukins’ appearance in Table 4 suggests that inflammatory mechanisms may mediate age-specific changes outside the brain (and /or non-dementing aspects of cognition). IL-6 has been reported to protect cognition in centenarians [44], but is not in TARCC's biomarker panel. The interleukin 1 receptor antagonist is related neither to age nor to δ (Table 6).

Eight of the eleven proteins we previously associated with δ in TARCC [13] are also in Class 2 (Table 5), including IFN-γ, Interleukins 10, 15, 12p40, and the Interleukin 1 receptor (IL-1r). Five of those eight [i.e., alpha2-macroglobulin (α2M), IFN-γ, IL-10, IL-12-p40 (IL-12p40), and SCF], exhibited otherwise significant trends in their associations with δ, which could not survive Bonferroni correction. Their previously reported associations were specific to NHW, while the current models were ethnicity adjusted. It remains to be seen whether ethnicity-specific effects on δ can be confirmed for any of the Class 2 proteins in Table 4.

Table 5 identifies many newly recognized age-independent determinants of dEQ (Class 3). Their relationships with δ are beyond the scope of this manuscript. However, GH's appearance on this list is of interest given the prominence of other somatomedins among the Class 1 and 2 proteins (Tables 2 and 4).

In summary, we have surveyed over 100 serum proteins for their possible roles as mediators of age's specific association with a latent dementia phenotype. 22 potential mediators were identified. These may offer targets for the disabling aspects of Aging Proper. An additional 41 age-related proteins were identified. These may mediate age's effects on other organs. Notable among them are the EGFR and many of its ligands. Some EGFR ligands may protect the brain and other organs from age-related changes. However, this may occur at a risk of incurring cancer. Conversely, the use of EGFR antagonists in cancer treatment may accelerate the effects of Aging Proper in the brain and other organs.

## METHODS

### Subjects

Subjects included n = 3385 TARCC participants, including 1240 cases of AD, 688 MCI cases, and 1384 NC. Each underwent serial annual standardized clinical examinations, culminating in a consensus clinical diagnosis of NC, MCI or AD. Institutional Review Board approval was obtained at each site and written informed consent was obtained from all participants.

δ's Indicators included Logical Memory II (LMII) [45], Visual Reproduction I (VRI) [45], the Controlled Oral Word Association (COWA) [46], Digit Span Test (DST) [45] and Instrumental Activities of Daily Living (IADL) [47]. All tests were available in Spanish translation. The indicators were not adjusted for this analysis. The resulting unadjusted δ homolog was validated by its association with dementia severity, as measured by the Clinical Dementia Rating Scale sum of boxes (CDR) [48] and by ROC analysis.

TARCC's methodology has been described elsewhere [49]. Serum samples were sent frozen to Rules-Based Medicine (RBM) in Austin, TX. There they were assayed without additional freeze-thaw cycles. RBM conducted multiplexed immunoassay via their human multi-analyte profile (human MAP). A complete listing of the biomarker panel we employed is available at http://www.rulesbasedmedicine.com.

Raw biomarker data were inspected to ascertain their normality. Data points beyond 3.0 standard deviations (SD) about the mean were labeled as “outliers” and deleted. Logarithmic transformation was used to normalize highly skewed distributions. The data were then standardized to a mean of zero and unit variance.

### Covariates

All observed measures in the structural models were adjusted for APOE ε4 burden, education, ethnicity, gender, HCY, and HgbA1c. Measurements of HCY, HgbA1c and APOE ε4 genotyping were performed in the Ballantyne laboratory at the Baylor College of Medicine. HgbA1c was measured in whole blood by the turbidimetric inhibition immunoassay (TINIA). HCY was measured in serum using the recombinant enzymatic cycling assay (i.e., Roche Hitachi 911).

### APOE genotyping

APOE genotyping was conducted using standard polymerase chain reaction (PCR) methods [50]. APOEε4 status was coded dichotmously based on the presence of one or more ε4 alleles. TARCC's RBM biomarkers exhibit significant batch effects. Therefore, each biomarker was additionally adjusted for dichotomous dummy variables coding batch.

### Statistical analysis

#### Analysis Sequence

This analysis was performed using Analysis of Moment Structures (AMOS) software [51]. The maximum likelihood estimator was chosen. All observed indicators were adjusted for age, education, ethnicity and gender. Co-variances between the residuals were estimated if they were significant and improved fit.

We used the ethnicity equivalent δ homolog (“dEQ”) as previously described [1]. That homolog has been reported to 1) have excellent fit (i.e., χ^2^/df = 181/24, p < 0.001; CFI = 0.97; RMSEA = 0.05), 2) have acceptable factor determinacy by Grice's Method [52], 3) exhibit factor equivalence across ethnicity, 4) to be strongly correlated with dementia severity as measured by the CDR (r = 0.99, p <0.001) and 5) to exhibit an AUC of 0.97 (CI: 0.97-0.98) for the discrimination between AD cases and controls (in Visit 2 TARCC data). For the purposes of this analysis, dEQ was again constructed in Visit 2 data, but without any covariates, specifically age, ethnicity, GDS, gender, HCY, HGbA1c and APOE ε4 status.

dEQ and g’ factor weights were applied to Visit 2 observed data to generate Visit 2 dEQ and g’ composite scores (i.e., dEQ v2 and g’ v2, respectively). g’ is dEQ's residual in Spearman's *g* [53]. The composite scores were used as observed dependent variables in multiple regression models of age's direct association with covariate adjusted Visit 2 g’ and dEQ.

Next, we constructed a longitudinal mediation model in SEM (Figure 1). Such models can arguably be interpreted causally [54]. Path “a” represents age's direct association with Visit 2 dEQ scores. Path “b” represents the Visit 1 biomarker's independent effect on dEQ. Bonferroni correction to p <0.001 was used to offset the potential for Type 2 error after multiple comparisons. When both paths were significant, we considered path “c”. The biomarker's mediation effect on age's direct association can then be calculated by MaKinnon's method [55].

The mediation models were constructed in a randomly selected subset of TARCC participants, comprising approximately 50% of the subjects (i.e., Class 1: n = 1691). As a test of each model's generalizability to the remainder (n = 1694), each mediation path's significant direct association was constrained across the two groups, and model fit compared across constrained and unconstrained conditions [56-57]. Mediation effects were calculated in the constrained models.

#### Missing data

We used the newest instance of TARCC's dataset (circa 2016). The entire dataset was employed. Clinical diagnoses were available on 3385 subjects, 2861 of whom had complete data for δ's cognitive indicators and covariates. Modern Missing Data Methods were automatically applied by the AMOS software [58]. AMOS employs Full information Maximum Likelihood (FIML) [59-60]. Only the ROC analyses, performed in Statistical Package for the Social Sciences (SPSS) [61], were limited to complete cases.

#### Fit indices

Fit was assessed using four common test statistics: chi-square, the ratio of the chi-square to the degrees of freedom in the model (CMIN /DF), the comparative fit index (CFI), and the root mean square error of approximation (RMSEA). A non-significant chi-square signifies that the data are consistent with the model [62]. However, in large samples, this metric conflicts with other fit indices (insensitive to sample size) show that the model fits the data very well. A CMIN/DF ratio < 5.0 suggests an adequate fit to the data [63]. The CFI statistic compares the specified model with a null model [64]. CFI values range from 0 to 1.0. Values below 0.90 suggest model misspecification. Values approaching 1.0 indicate adequate to excellent fit. An RMSEA of 0.05 or less indicates a close fit to the data, with models below 0.05 considered “good” fit, and up to 0.08 as “acceptable“ [65]. All fit statistics should be simultaneously considered when assessing the adequacy of the models to the data.
